# Supplementary material for: Tumor cell-adipocyte gap junctions activate lipolysis and contribute to breast tumorigenesis
Source: Nat Commun. 2025 Aug 20;16:7438. doi: 10.1038/s41467-025-62486-3 (PMC12368202; doi:10.1038/s41467-025-62486-3)
Supplement: Supplementary file 1 — Supplementary Information [file 41467_2025_62486_MOESM1_ESM.pdf]

Supplementary Information for

Tumor cell-adipocyte gap junctions activate lipolysis and contribute to breast tumorigenesis

Jeremy Williams[1,2 †], Roman Camarda[1,2 †], Serghei Malkov[3], Lisa J. Zimmerman[4,5], Suzanne Manning[6], Dvir Aran[7,8], Andrew Beardsley[1,9], Daniel Van de Mark[1], Rachel Nakagawa[1,2], Yong Chen[1,10,11,12], Charles Berdan[13,14,15], Sharon M. Louie[13,14,15], Celine Mahieu[1], Daphne Superville[1,2], Juliane Winkler[16,17,18], Elizabeth Willey[16,17], Erica J. Hutchins[1,11], John D. Gagnon[2,19,20], Seda Kilinc Avsaroglu[1], Kosaku Shinoda[1,10,11,21], Matthew Gruner[1], Hiroshi Nishida[22,23], K. Mark Ansel[19,20], Zena Werb[16,17], Daniel K. Nomura[13,14,15], Shingo Kajimura[1,10,11,22,23], Atul J. Butte[7], Melinda E. Sanders[6], Daniel C. Liebler[4,5], Hope Rugo[9,24], Gregor Krings[25], John A. Shepherd[26], and Andrei Goga[1,9,17*]

[1]Department of Cell & Tissue Biology, University of California, San Francisco, San Francisco, CA, USA.
[2]Biomedical Sciences Graduate Program, University of California, San Francisco, San Francisco, CA, USA.
[3]Department of Radiology & Biomedical Imaging, University of California, San Francisco, San Francisco, CA, USA.
[4]Department of Biochemistry, Vanderbilt University School of Medicine, Nashville, TN, USA.
[5]Jim Ayers Institute for Precancer Detection and Diagnosis, Vanderbilt-Ingram Cancer Center, Nashville, TN, USA.
[6]Department of Pathology, Vanderbilt University School of Medicine, Nashville, TN, USA.
[7]Faculty of Biology, Technion, Israel Institute of Technology, Haifa, Israel.
[8]The Taub Faculty of Computer Science, Technion, Israel Institute of Technology, Haifa, Israel.
[9]Department of Medicine, University of California, San Francisco, San Francisco, CA, USA.
[10]Diabetes Center, University of California, San Francisco, San Francisco, CA, USA.
[11]Eli and Edythe Broad Center of Regeneration Medicine and Stem Cell Research, University of California, San Francisco, San Francisco, CA, USA.
[12]Tongji Medical College, Huazhong University of Science and Technology, Wuhan, China.
[13]Department of Chemistry, University of California, Berkeley, Berkeley, CA, USA.
[14]Department of Molecular & Cell Biology, University of California, Berkeley, Berkeley, CA, USA.
[15]Department of Nutritional Sciences & Toxicology, University of California, Berkeley, Berkeley, CA, USA.
[16]Department of Anatomy, University of California, San Francisco, San Francisco, CA, USA.
[17]Helen Diller Family Comprehensive Cancer Center, University of California, San Francisco, San Francisco, CA, USA.
[18]Center for Cancer Research, Medical University of Vienna, Austria.
[19]Department of Microbiology & Immunology, University of California, San Francisco, San Francisco, CA, USA.
[20]Sandler Asthma Basic Research Center, University of California, San Francisco, San Francisco, CA, USA.
[21]Department of Medicine and Molecular Pharmacology, Albert Einstein College of Medicine, Bronx, NY, USA.
[22]Division of Endocrinology, Diabetes and Metabolism, Beth Israel Deaconess Medical Center, Harvard Medical School, Boston, MA, USA.
[23]Howard Hughes Medical Institute, Chevy Chase, MD USA.
[24]Department of Medical Oncology & Therapeutics Research, City of Hope Comprehensive Cancer Center, Duarte, CA, USA.
[25]Department of Pathology, University of California, San Francisco, San Francisco, CA, USA.
[26]Cancer Center, University of Hawaii, Honolulu, HI, USA.

[†]These authors contributed equally

*Correspondence to: Andrei.Goga@ucsf.edu

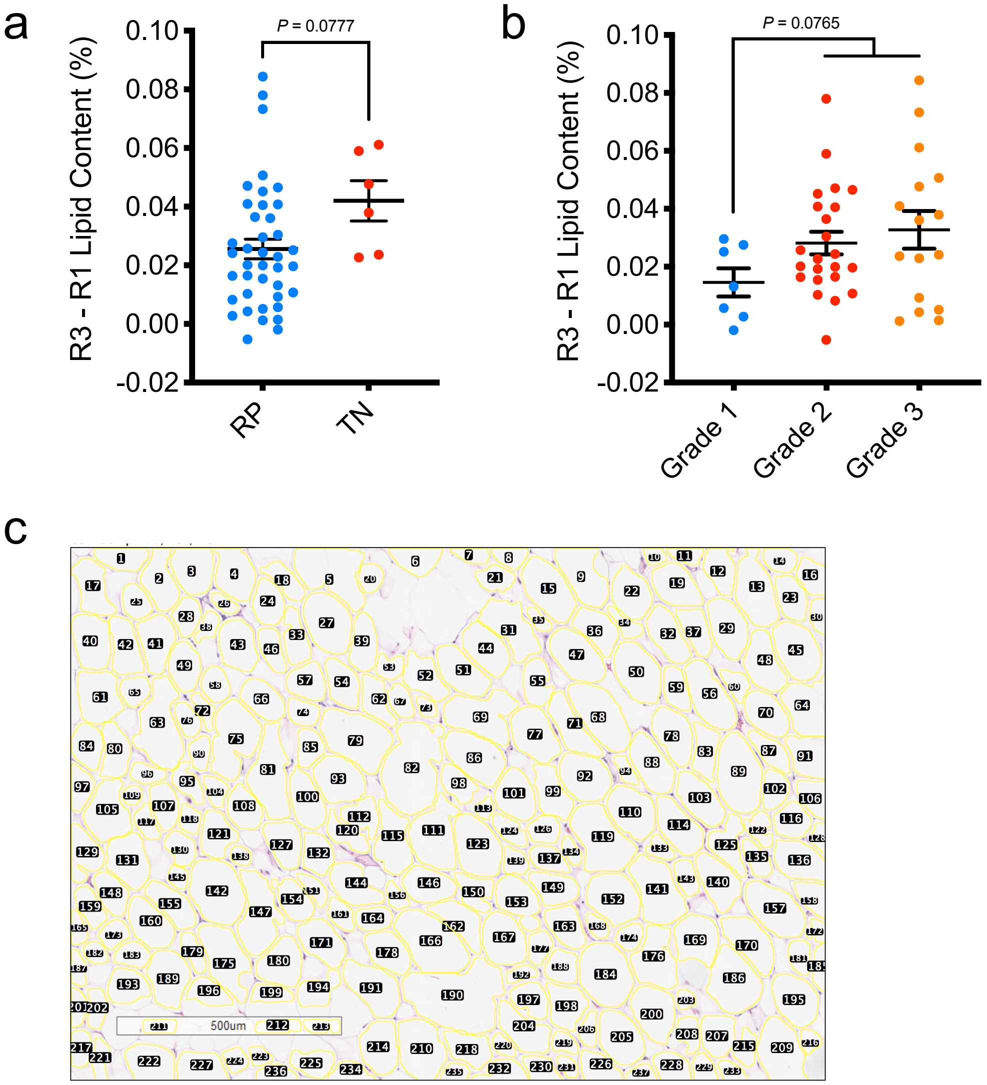

Supplementary Fig. 1: NAT lipid content by receptor status and tumor grade, and adipocyte area quantification. a Change in lipid content in R3 of NAT versus R1 of NAT from TN (n = 6) and RP (n = 40) patients. **b** Change in lipid content in R3 of NAT versus R1 of NAT from grade 1 (n = 7), 2 (n = 23) and 3 (n = 16) patients. **c** Example of Adiposoft software output on manual mode before curation to identify whole, individual adipocytes. Represantative image from analysis performed on samples from multiple regions surrounding (n = 11) patient breast tumors. P values indicated; unpaired two-tailed t test **a** and **b**. Source data are provided as a Source Data file.

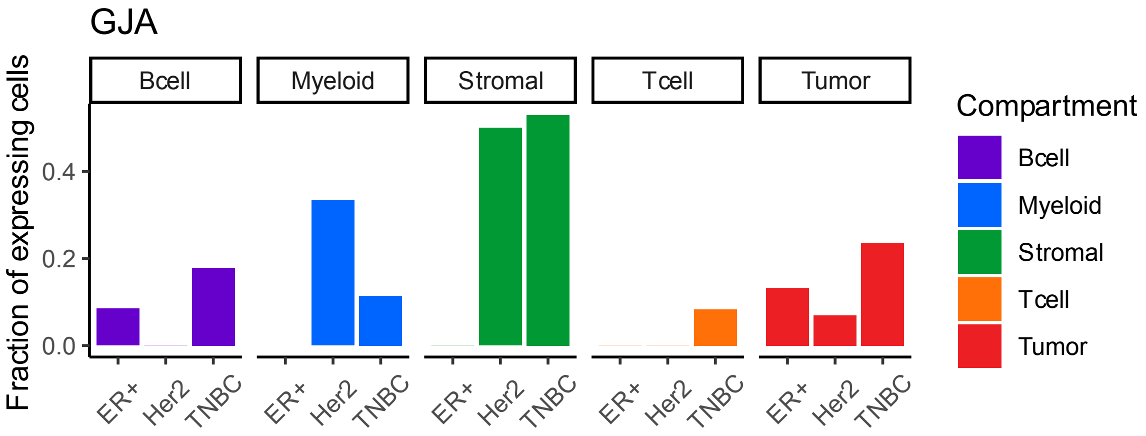
GJA
Bcell
Myeloid
Stromal
Tcell
Tumor
Fraction of expressing cells
0.4
0.2
0.0
ER+
Her2
TNBC
Compartment
Bcell
Myeloid
Stromal
Tcell
Tumor

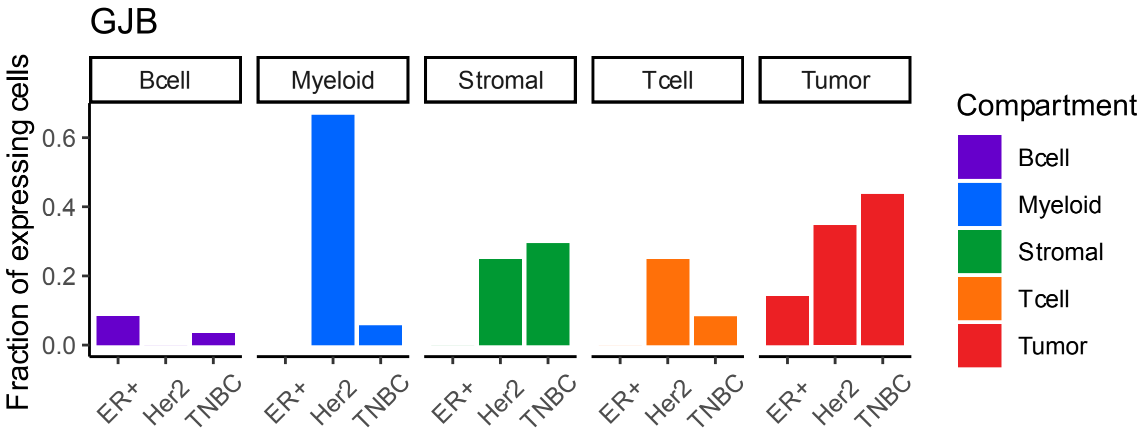
GJB
Bcell
Myeloid
Stromal
Tcell
Tumor
Fraction of expressing cells
0.6
0.4
0.2
0.0
ER+
Her2
TNBC
Compartment
Bcell
Myeloid
Stromal
Tcell
Tumor

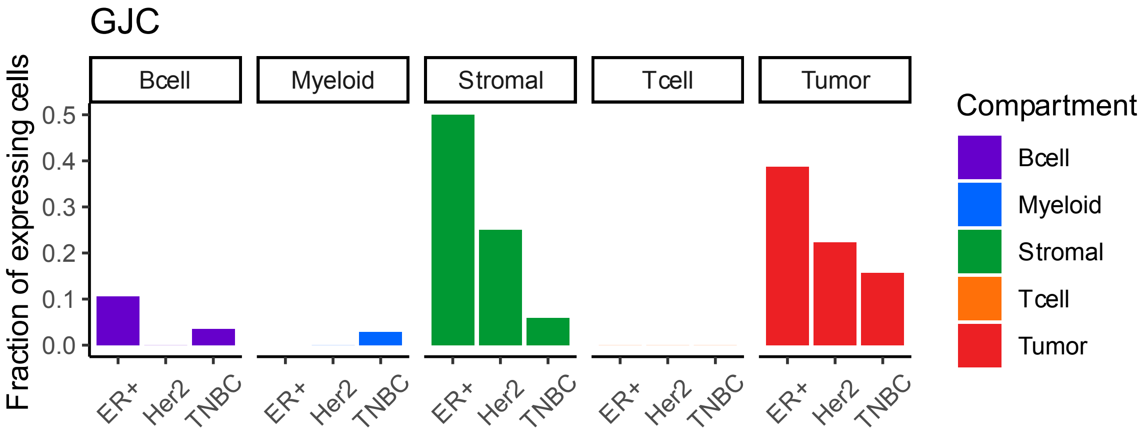
GJC
Bcell
Myeloid
Stromal
Tcell
Tumor
Fraction of expressing cells
0.5
0.4
0.3
0.2
0.1
0.0
ER+
Her2
TNBC
Compartment
Bcell
Myeloid
Stromal
Tcell
Tumor

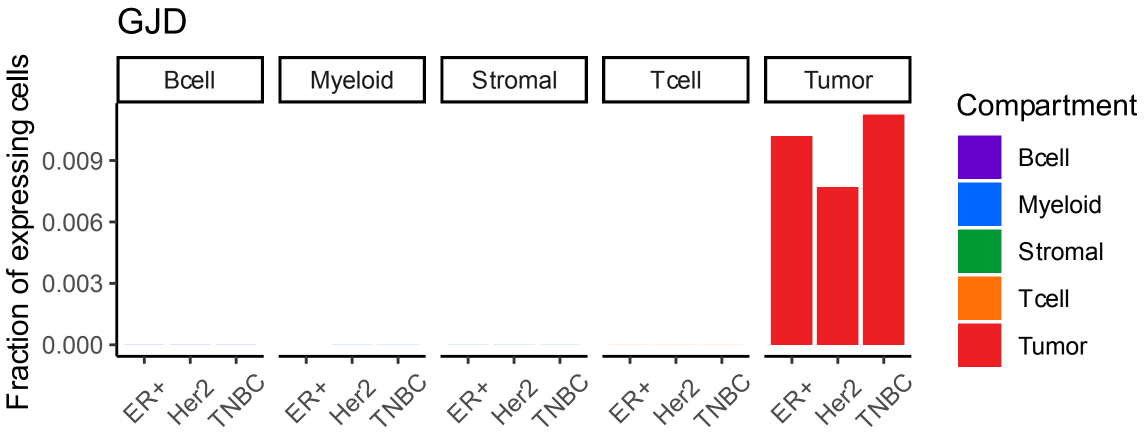
GJD
Bcell
Myeloid
Stromal
Tcell
Tumor
Fraction of expressing cells
0.009
0.006
0.003
0.000
ER+
Her2
TNBC
Compartment
Bcell
Myeloid
Stromal
Tcell
Tumor

Supplementary Fig. 2: Fraction of cells expressing gap junction family by tumor compartment cell type. Single cell (n = 515 cells) RNA-seq of B cell (n = 83), myeloid cell (n = 38), stromal cell (n = 23), T cell (n = 54) and tumor (n = 317) cell compartments from the patient (n = 11) tumor microenvironment. Source data are provided as a Source Data file.

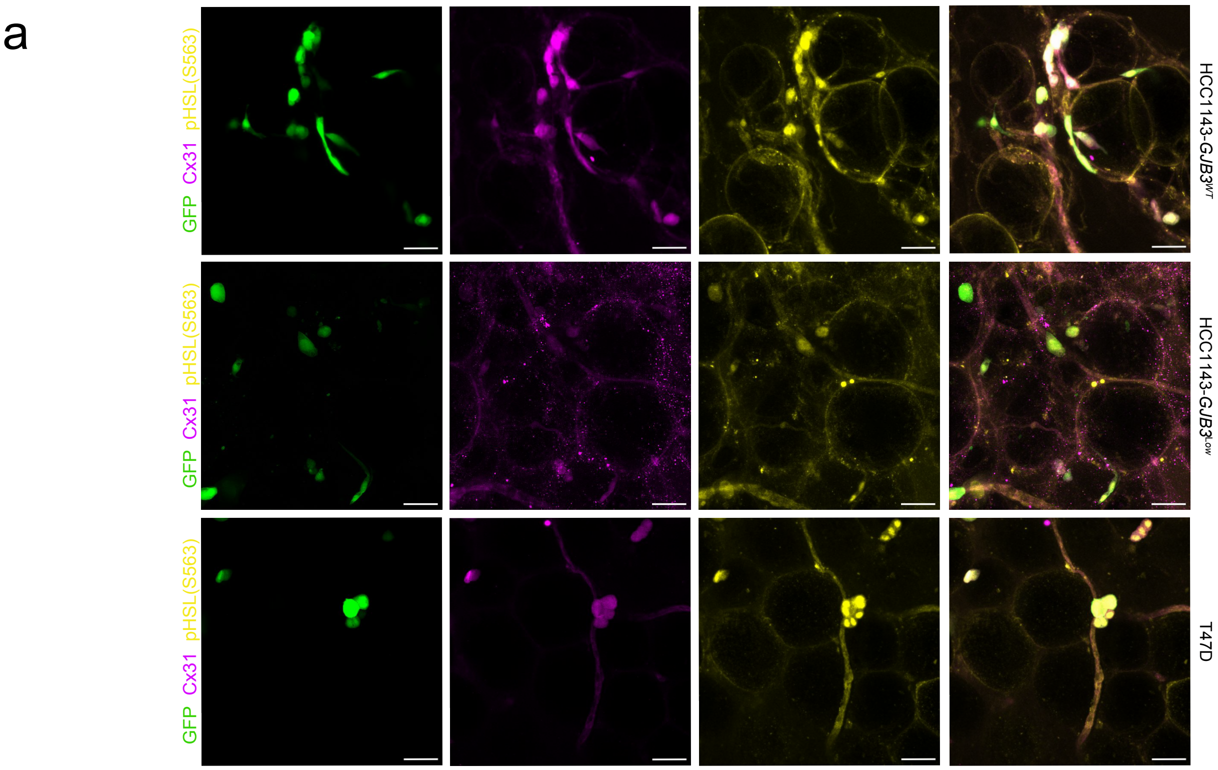

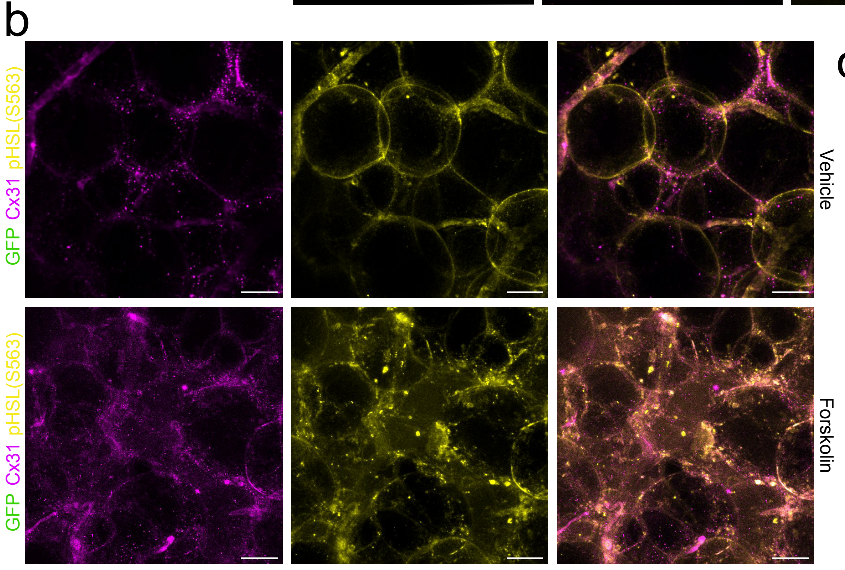

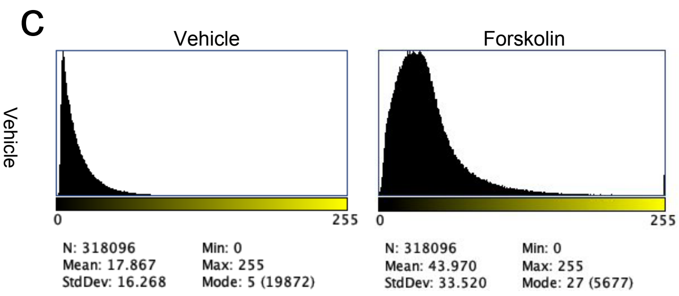

d

Control	No. of Puncta	Percent Area
Vehicle	13	0.016
Forskolin	78	0.069

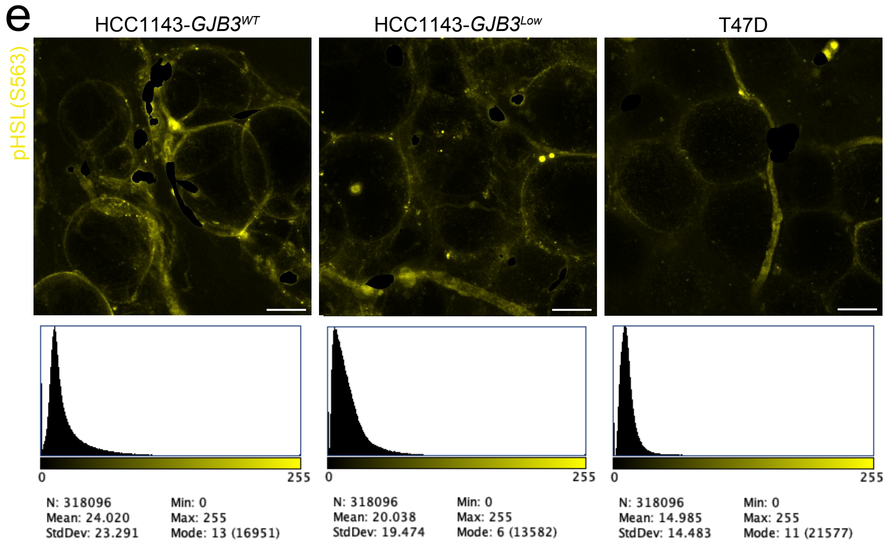

f

Co-cultured Line	No. of Puncta	Percent Area
HCC1143-*GJB3*^{WT}	21	0.026
HCC1143-*GJB3*^{Low}	11	0.016
T47D	4	0.004

Supplementary Fig. 3: Quantification of lipolysis signaling in primary mammary adipose tissue from indicated cancer cell co-cultures and controls. a Staining with Cx31 (magenta) pHSL(S563) (yellow) of primary mammary tissue from a healthy individual (PT003) injected with GFP-expressing HCC1143-*GJB3*^{WT} (top), HCC1143-*GJB3*^{Low} (middle), or T47D cells (bottom) and co-cultured overnight. White arrowheads indicate co-staining of Cx31 and pHSL(S563) at contact point between GFP cancer cells and adipocytes. Scale bar, 50 µm. **b** Staining with Cx31 (magenta) and pHSL(S563) (yellow) of control mammary adipose tissue injected with either vehicle (top) or 10 µM forskolin (bottom) and cultured for 24 hours. Scale bar, 50 µm. **c** Histogram of pHSL(S563) expression in indicated co-culture control tissues. **d** Quantification of pHSL(S563) puncta number and percent total area of puncta in indicated co-culture control tissue images from **b**. **e** Staining (top) and histogram (bottom) of pHSL(S563) in mammary adipose tissue co-cultured with indicated GFP-tagged breast cancer cell lines. With images from A, cancer cell pHSL(S563) signal was masked out using GFP tag. Scale bar, 50 µm. **f** Quantification of pHSL(S563) puncta number and percent total area of puncta in mammary adipose tissue co-cultured with indicated cell line from **e**. Co-culture and control images are representative from experiments repeated in 3 independent biological replicates.

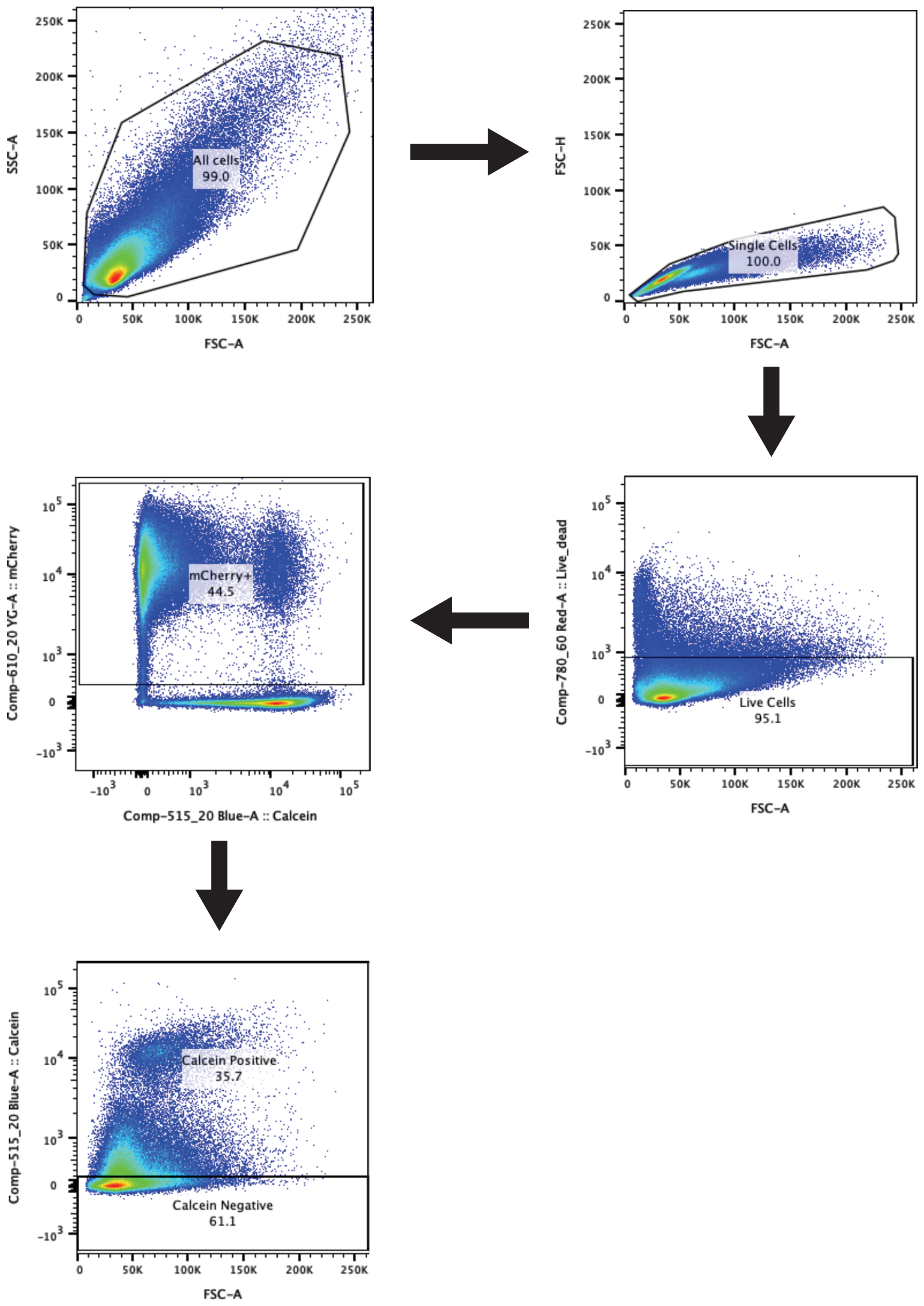

Supplementary Fig. 4: Flow cytometry gating strategy to identify mCherry-positive, CalceinAM-positive cells. Representative experimental control sample, HCC1143 cells. Side scatter and forward scatter were used to distinguish all cells from debris. Forward scatter was used to distinguish singlets (single cells) from all cells. Live cells were identified as negative for live/dead staining. Live single cells positive for mCherry were identified. Of mCherry-positive cells, CalceinAM-positive and –negative populations were distinguished. Representative figures shown from (n = 3) biological replicates; final panel represents gating for Figs. 3a and Fig. 3b.

Supplementary Table 1: Sample specifications and study sources for applied clinical samples and datasets.

<table>
<tr><th>Figure/Table</th><th>Study Name/Title</th><th>Author Name</th><th>Sample Type</th><th>Data Collected</th><th colspan="18">Number of Samples</th><th>Reference no./ Citation</th></tr>
<tr><td rowspan="3">Fig. 1a-d
Supplementary Figure 1
Supplementary Data 1</td><td rowspan="3">Mammographic quantitative image analysis and biologic image composition for breast lesion characterization and classification</td><td rowspan="3">Drukker et al. 2014</td><td rowspan="3">Breast mammography imagery of primary lesion</td><td rowspan="3">3CB</td><td colspan="10">3CB</td><td colspan="4">Pathology</td><td colspan="4">RCB</td><td rowspan="3">Reference no. 17</td></tr>
<tr><td colspan="5">Triple-Negative</td><td colspan="5">Receptor-Positive</td><td colspan="4" rowspan="2">11/46</td><td colspan="4" rowspan="2">Unknown</td></tr>
<tr><td colspan="5">6</td><td colspan="5">40</td></tr>
<tr><td rowspan="2">Fig. 1e</td><td rowspan="2">Epithelial progeny of estrogen-exposed breast progenitor cells display a cancer-like methylome</td><td rowspan="2">Cheng et al. 2008</td><td rowspan="2">Microdissection of tissues from patient mastectomy</td><td rowspan="2">IHC on primary breast lesion and surrounding tissue by radius, contralateral and normal tissue</td><td colspan="3">Primary Tumor</td><td colspan="3">NAT 1cm</td><td colspan="3">NAT 2cm</td><td colspan="3">NAT 3cm</td><td colspan="3">NAT 4Cm</td><td colspan="3">Healthy NT</td><td rowspan="2">Reference no. 20</td></tr>
<tr><td colspan="3">9</td><td colspan="3">7</td><td colspan="3">5</td><td colspan="3">3</td><td colspan="3">4</td><td colspan="3">10</td></tr>
<tr><td rowspan="2">Supplementary Data 2
Fig. 1f</td><td rowspan="2">Data first reported here</td><td rowspan="2">Williams/ Camarda et al. 2025</td><td rowspan="2">Laser capture microdissection of primary patient tumors and adjacent tissue, and healthy patient control tissue.</td><td rowspan="2">LC-MS/MS</td><td colspan="2">Healthy Control</td><td colspan="2">NAT</td><td colspan="2">Stroma</td><td colspan="2">Lum.A</td><td colspan="2">Lum.B</td><td colspan="2">Lum.A/ B</td><td colspan="2">Her2-Amp</td><td colspan="2">Her2-Amp/ Lum.B</td><td colspan="2">Basal</td><td rowspan="2">First reported here</td></tr>
<tr><td colspan="2">42</td><td colspan="2">4</td><td colspan="2">36</td><td colspan="2">38</td><td colspan="2">16</td><td colspan="2">1</td><td colspan="2">9</td><td colspan="2">5</td><td colspan="2">16</td></tr>
<tr><td rowspan="2">Fig. 2d</td><td rowspan="2">The Cancer Genome Atlas Program</td><td rowspan="2">TCGA</td><td rowspan="2">Primary breast cancer lesion tissue</td><td rowspan="2">RNA-Seq</td><td colspan="9">Triple-Negative</td><td colspan="9">Receptor-Positive</td><td rowspan="2">TCGA Research Network, https://www.cancer.gov/ccg/research/genome-sequencing/tcga</td></tr>
<tr><td colspan="9">123</td><td colspan="9">648</td></tr>
<tr><td>Fig. 2e
Supplementary Figure 2</td><td>Single-cell RNA-seq enables comprehensive tumour and immune cell profiling in primary breast cancer</td><td>Chung et al. 2017</td><td>Primary breast cancer lesion tissue</td><td>scRNA-Seq</td><td colspan="18">317 cells total from 11 tumors were sequenced</td><td>Reference no. 38</td></tr>
<tr><td>Fig. 3
Supplementary Figure 3</td><td>Data first reported here</td><td>Williams/ Camarda et al. 2025</td><td>Primary breast adipose tissue from healthy individuals, Werb Lab, UCSF</td><td>IHC, IF</td><td colspan="18">9 samples prefixed ‘PT’</td><td>First reported here</td></tr>
</table>
