# Supplementary material for: Tumor cell-adipocyte gap junctions activate lipolysis and contribute to breast tumorigenesis
Source: Nat Commun. 2025 Aug 20;16:7438. doi: 10.1038/s41467-025-62486-3 (PMC12368202; doi:10.1038/s41467-025-62486-3)
Supplement: Supplementary file 2 — Description of Additional Supplementary Files [file 41467_2025_62486_MOESM2_ESM.pdf]

Description of Additional Supplementary Files:

Supplementary Data 1: Patient ID, receptor status, histological section availability, percent lipid content (lipid content / lipid + water + protein content) of L, R1, R2 and R3, and Scarff-Bloom-Richardson (SBR) grade from patients (n = 46) with invasive breast cancer.

Supplementary Data 2: LC-MS/MS of LCM samples from 75 patients with invasive breast cancer and 42 healthy subjects. a Sample number, ID number, tissue type, and tumor subtype (when applicable) of 75 patients and 42 healthy subjects. b Normalized precursor ion intensities for proteins detected via LC-MS/MS from samples in a.

Supplementary Data 3: RNA expression changes in MTB-TOM tumors (n = 10) compared to non-tumor mammary glands (n = 3). Differential expression analysis was performed using the ‘limma’ R package.
