# Supplementary material for: Tumor cell-adipocyte gap junctions activate lipolysis and contribute to breast tumorigenesis
Source: Nat Commun. 2025 Aug 20;16:7438. doi: 10.1038/s41467-025-62486-3 (PMC12368202; doi:10.1038/s41467-025-62486-3)
Supplement: Supplementary file 6 — Reporting Summary [file 41467_2025_62486_MOESM6_ESM.pdf]

nature portfolio

Corresponding author(s): Andrei Goga

Last updated by author(s): May 2, 2025

Reporting Summary

Nature Portfolio wishes to improve the reproducibility of the work that we publish. This form provides structure for consistency and transparency in reporting. For further information on Nature Portfolio policies, see our Editorial Policies and the Editorial Policy Checklist.

Statistics

For all statistical analyses, confirm that the following items are present in the figure legend, table legend, main text, or Methods section.

n/a	Confirmed	
☐	☒	The exact sample size (n) for each experimental group/condition, given as a discrete number and unit of measurement
☐	☒	A statement on whether measurements were taken from distinct samples or whether the same sample was measured repeatedly
☐	☒	The statistical test(s) used AND whether they are one- or two-sided<br>*Only common tests should be described solely by name; describe more complex techniques in the Methods section.*
☒	☐	A description of all covariates tested
☐	☒	A description of any assumptions or corrections, such as tests of normality and adjustment for multiple comparisons
☐	☒	A full description of the statistical parameters including central tendency (e.g. means) or other basic estimates (e.g. regression coefficient) AND variation (e.g. standard deviation) or associated estimates of uncertainty (e.g. confidence intervals)
☒	☐	For null hypothesis testing, the test statistic (e.g. F, t, r) with confidence intervals, effect sizes, degrees of freedom and P value noted<br>*Give P values as exact values whenever suitable.*
☒	☐	For Bayesian analysis, information on the choice of priors and Markov chain Monte Carlo settings
☒	☐	For hierarchical and complex designs, identification of the appropriate level for tests and full reporting of outcomes
☐	☒	Estimates of effect sizes (e.g. Cohen's d, Pearson's r), indicating how they were calculated

Our web collection on statistics for biologists contains articles on many of the points above.

Software and code

Policy information about availability of computer code

Data collection	Publicly available data sets were acquired as noted in the text. Our annotations of the TCGA data set are available at (https://bitbucket.org/jeevb/brca).
Data analysis	Prism software (v 10.4.1) was used to generate and analyze Spearman correlation (Fig. 1d) and the survival plots (Figs. 4b, 4c and 4g). Survival plot P-values were generated using a log-rank test. Correlation P values were generated using ordinary one-way ANOVA with multiple comparisons (Figs. 1f, 1h, 2b, 3f, 3h, 4a right, 4d and 4f), two-way ANOVA with multiple comparisons (Fig. 1c), repeated measures one-way ANOVA with multiple comparisons (Figs. 1b, 3e, and 3g), repeated measures mixed effects model with multiple comparisons (Fig. 1e), and unpaired two-tailed t test (Figs. 2a, 2c, 4a left and center, 4e, and Supplementary Fig. 1a-b). These analyses were performed using PRISM software. DGE analysis of TN compared to RP patient tumors (Fig. 2d) was calculated using the 'limma' R package (reference 62 in the manuscript). DGE analysis of MTB-TOM compared to normal mammary gland (Fig. 2f) was performed using the DESeq2 package (reference 63 in the manuscript). Differential expression analyses (Figs. 2d and 2f) were calculated with a false discovery rate of 0.05. Biological replicates are shown in Figs. 1b-e, 1g-h, 2a-c, 3e-h, 4a, 4c-f, and Supplementary Figs. 1a-b. Technical duplicates for each biological replicate are shown in Fig. 1f. Immunofluorescence images shown in Figs. 3a-b, 3d, and Supplementary Figs. 3a-b and 3e are representative from experiments repeated in 3 independent biological replicates.

For manuscripts utilizing custom algorithms or software that are central to the research but not yet described in published literature, software must be made available to editors and reviewers. We strongly encourage code deposition in a community repository (e.g. GitHub). See the Nature Portfolio guidelines for submitting code & software for further information.

Data

Policy information about availability of data

All manuscripts must include a data availability statement. This statement should provide the following information, where applicable:

- Accession codes, unique identifiers, or web links for publicly available datasets
- A description of any restrictions on data availability
- For clinical datasets or third party data, please ensure that the statement adheres to our policy

For Figure 1e, we used RNAseq data from reference 20, which has been deposited in ArrayExpress (http://www.ebi.ac.uk/arrayexpress/) under accession code E-E-TABM-276, and RNAseq data from reference 19, which has been deposited in Array Express under accession code E-MTAB-2602. For Figure 1f, we generated protein MS data which has been deposited in ProteomeXchange [https://proteomecentral.proteomexchange.org/] under accession code MSV000097890. For Figure 2d, we used data downloaded from the Cancer Genome Browser (https://cancergenome.nih.gov), which is available under accession code phs000178 and our annotations for the data are available at (https://bitbucket.org/jeevb/brca). For Figs. 2e and Supplementary Fig. 2, we used scRNA-seq data from reference 38, which has been deposited in the NCBI Gene Expression Omnibus database under the accession code GSE75688. For Figure 2f, we used RNAseq data reference 71, which is available in the NCBI Gene Expression Omnibus database under the accession code GSE130921. Source data are provided as a Source Data file.

Research involving human participants, their data, or biological material

Policy information about studies with human participants or human data. See also policy information about sex, gender (identity/presentation), and sexual orientation and race, ethnicity and racism.

Reporting on sex and gender	Sex information for patient data presented in Figs. 1b-d and Supplementary Data 1 (n=46 biologically female patients) was previously published (reference 17 in the manuscript); gender information was not obtained for analyses presented in this study. For Fig. 1f (n=75 patients with invasive breast cancer and 42 healthy patients), data from biologically female patients was used, and information on patient gender was not collected. Information on biological sex for assays involving human biological material in Figs. 3b,d,g,e and Supplementary Figure 3 is available: biological material were from (n=9) biological women; gender information was not considered in these assays and analyses as gender was not reported in available patient data. All patient data and biological samples presented in this work are anonymized. The World Health Organization estimates that 99% of all breast cancers occur in biological females; Results in these studies apply predominately to biological females, and to all genders.
Reporting on race, ethnicity, or other socially relevant groupings	This study does not present information reporting on race, ethnicity or other socially relevant groupings. Reporting on these groupings was not available in the primary or published patient data or applied within this manuscript.
Population characteristics	Patient data presented in Figs. 1b-d and Supplementary Data 1 was from a previously published study (Drukker, ref. 17), and patient population characteristics were not made available for analyses presented in this study; Biological women with suspicious findings at mammography were recruited for imaging in the referenced pilot study. For Fig. 1F; laser capture microdissection was performed on tumors from 75 biological women with invasive breast cancer and on non-tumor breast tissue from 40 healthy biological females; population characteristics were not reported for those samples. For human biological material in Figs. 3b and 3d population characteristics data was not made available for any analyses; patient mammary fat samples utilized in these assays were tissues from healthy biological women who underwent elective breast reduction surgery.
Recruitment	No patient recruitment was performed for primary data presented within this study. Patient data presented in Figs. 1b-d and Supplementary Data 1 was from a previously published study (Drukker, ref. 17); all study participants provided written informed consent and received compensation for any imaging conducted in addition to the standard of care they were receiving.
Ethics oversight	Collection of all patient data and human biological materials was approved by the UCSF Institutional Review Board.

Note that full information on the approval of the study protocol must also be provided in the manuscript.

Field-specific reporting

Please select the one below that is the best fit for your research. If you are not sure, read the appropriate sections before making your selection.

[x] Life sciences [ ] Behavioural & social sciences [ ] Ecological, evolutionary & environmental sciences

For a reference copy of the document with all sections, see nature.com/documents/nr-reporting-summary-flat.pdf

Life sciences study design

All studies must disclose on these points even when the disclosure is negative.

Sample size	No statistical method was used to predetermine sample size. The sample size for all experiments (in vitro and in vivo) was not chosen with consideration of adequate power to detect a prespecified effect size. The sample sizes vary and are sufficient based upon the inherent variability of each methodology utilized.
Data exclusions	No data were excluded from the analyses.

Replication	All attempts at replication were successful.
Randomization	For all in vivo studies, mice were randomized to treatment groups when tumors reached a predetermined volume, or palpability, on a per experiment basis.
Blinding	The investigators were not blinded to group allocation during data collection and analysis for in vivo experiments. The investigators were blinded during immunofluorescence analysis. In most cases treatment studies were carried out by a single researcher, such that blinding was not technically feasible.

Reporting for specific materials, systems and methods

We require information from authors about some types of materials, experimental systems and methods used in many studies. Here, indicate whether each material, system or method listed is relevant to your study. If you are not sure if a list item applies to your research, read the appropriate section before selecting a response.

Materials & experimental systems

n/a	Involved in the study	
☐	☒	Antibodies
☐	☒	Eukaryotic cell lines
☒	☐	Palaeontology and archaeology
☐	☒	Animals and other organisms
☒	☐	Clinical data
☒	☐	Dual use research of concern
☒	☐	Plants

Methods

n/a	Involved in the study	
☒	☐	ChIP-seq
☐	☒	Flow cytometry
☒	☐	MRI-based neuroimaging

Antibodies

Antibodies used

The primary antibodies targeting the following proteins were used. Immunoblot: β-actin (actin) (sc-47778 HRP, Santa Cruz, 1:10,000), pHSL S563 (4139, Cell Signaling, 1:1000), HSL (4107, Cell Signaling, 1:1000), HNF4a (ab41898, Abcam, 1:1000), and Cx31 (ab236620, Abcam, 1:1000). Immunofluorescence: pan-cytokeratin (sc-81714, Santa Cruz, 1:50), pHSL-S563 (4139, Cell Sig, 1:100), Cx31 (rabbit, 12880-1-AP, Proteintech, 1:50), and Cx31 (mouse, WH0002707M1, Sigma, 1:100).

Validation

All antibodies have been validated by manufacturer:
Immunoblot:
β-actin (actin) (sc-47778 HRP, Santa Cruz):
https://www.scbt.com/p/beta-actin-antibody-c4?srsltid=AfmBOop6XjPinfuXbKX6ip_8Wh5n6hmgNqOtslLG95MzOdPLkEf5lMQN

pHSL S563 (4139, Cell Signaling):
https://www.cellsignal.com/products/primary-antibodies/phospho-hsl-ser563-antibody/4139?
srsltid=AfmBOoqolT_WNawyEoQQTGSIzAd93GSAGaJFaJFeUBdk5H_yXCGuJ8e

HSL (4107, Cell Signaling):
https://www.cellsignal.com/products/primary-antibodies/hsl-antibody/4107?
srsltid=AfmBOooXamKgi6IguiaWv7YCo_ylN79WbBzya2Nl5OljvDXYzKRfX6qO

HNF4a (ab41898, Abcam):
https://www.abcam.com/en-us/products/primary-antibodies/hnf-4-alpha-antibody-k9218-ab41898?srsltid=AfmBOoqCgAY-GP0BtFyhz0CcFKzhaLR0tHKx-NSxJ6dEHjxEnjyB1Zua#overlay=images

Cx31 (ab236620, Abcam):
https://doc.abcam.com/datasheets/inactive/ab236620/en-us/gjb3-cx31-antibody-ab236620.pdf

Immunofluorescence:
pan-cytokeratin (sc-81714, Santa Cruz):
https://www.scbt.com/p/pan-cytokeratin-antibody-ae1-ae3?
srsltid=AfmBOop5mGE6DKlAas2pWTrygNxwA1LqFwxvElyVBwvdywKBBBLVCT9A

pHSL-S563 (4139, Cell Signaling):
https://www.cellsignal.com/products/primary-antibodies/phospho-hsl-ser563-antibody/4139?
srsltid=AfmBOooZ7vVCtgbLPgl0zPmrEyQ4mSmI5uKy1cmAJunW2Ctg82KLndC

Cx31 (12880-1-AP, Proteintech):
https://www.ptglab.com/products/GJB3-Antibody-12880-1-AP.htm?
srsltid=AfmBOor681xrVK63I7JN9UcImh0VgC4JHe2ctY7pai8cNuvBrcLK2FyZ

Cx31 (WH0002707M1, Sigma):
https://www.sigmaaldrich.com/US/en/product/sigma/wh0002707m1?srsltid=AfmBOopFMpBqskMhWh2puAYdhagW9h2ps9RL6C0hWBSHxCUtQAp4znVB

Eukaryotic cell lines

Policy information about cell lines and Sex and Gender in Research

Cell line source(s)	ATCC: HCC1428 (HCC1428 - CRL-2327), T47D (T-47D - HTB-133), HCC3153 (CVCL_3377), HS578T (Hs 578T - HTB-126), BT549 (BT-549 - HTB-122) and HCC1143 (HCC1143 - CRL-2321) were derived from the primary breast cancer cells of female patients and were originally obtained from the collection of Dr. Adi Gazdar at UT Southwestern Medical Center, or from ATCC.
Authentication	Authenticated by ATCC and by independent STR profiling.
Mycoplasma contamination	All cell lines tested negative for mycoplasma contamination.
Commonly misidentified lines (See ICLAC register)	No commonly misidentified lines were used in this study.

Animals and other research organisms

Policy information about studies involving animals; ARRIVE guidelines recommended for reporting animal research, and Sex and Gender in Research

Laboratory animals	Four-week-old female WT FVB/N mice (Taconic FVB-F) and immunocompromised NOD/SCID-gamma (NSG) mice (Taconic NODSC) were purchased from Taconic Biosciences.
Wild animals	The study did not involve wild animals.
Reporting on sex	Female mice were used in these studies; The World Health Organization estimates that 99% of all breast cancers occur in biological females.
Field-collected samples	The study did not involve field-collected samples.
Ethics oversight	All in vivo mouse studies presented in this manuscript were conducted under protocol approved by the UCSF Institutional Animal Care & Use Committee (IACUC).

Note that full information on the approval of the study protocol must also be provided in the manuscript.

Plants

Seed stocks	*Report on the source of all seed stocks or other plant material used. If applicable, state the seed stock centre and catalogue number. If plant specimens were collected from the field, describe the collection location, date and sampling procedures.*
Novel plant genotypes	*Describe the methods by which all novel plant genotypes were produced. This includes those generated by transgenic approaches, gene editing, chemical/radiation-based mutagenesis and hybridization. For transgenic lines, describe the transformation method, the number of independent lines analyzed and the generation upon which experiments were performed. For gene-edited lines, describe the editor used, the endogenous sequence targeted for editing, the targeting guide RNA sequence (if applicable) and how the editor was applied.*
Authentication	*Describe any authentication procedures for each seed stock used or novel genotype generated. Describe any experiments used to assess the effect of a mutation and, where applicable, how potential secondary effects (e.g. second site T-DNA insertions, mosiacism, off-target gene editing) were examined.*

Flow Cytometry

Plots

Confirm that:

- [x] The axis labels state the marker and fluorochrome used (e.g. CD4-FITC).
- [x] The axis scales are clearly visible. Include numbers along axes only for bottom left plot of group (a 'group' is an analysis of identical markers).
- [x] All plots are contour plots with outliers or pseudocolor plots.
- [x] A numerical value for number of cells or percentage (with statistics) is provided.

Methodology

Sample preparation	For cancer cell-cancer cell transfer, monolayers of indicated lines (donors) were labelled with 1µM CalceinAM dye (Life Technologies) at 37°C for 40 min. Dye-loaded cells were washed three times with PBS, and then single-cell suspensions of 1.5

	X 105 mCherry-labelled cells (recipients) were added for 5 hours. For CBX treatment studies, monolayers of indicated lines (recipients) were pre-treated for 24 hours with 150uM CBX or vehicle. Indicated 'donor' cells were loaded in suspension with CalceinAM dye (Life Technologies) at 37°C for 40min, washed three times with PBS, and added onto indicated 'recipient' cells for 5 hours. Dye transfer was quantified by BD LSRFORTESSA or BD LSR II (BD Biosciences).
Instrument	BD LSRFORTESSA or BD LSR II
Software	Data was collected using BD software (FACSDIVA) and analyzed using FlowJo.
Cell population abundance	Our studies did not involve post-sort fractions.
Gating strategy	Side scatter and forward scatter were used to distinguish all cells from debris. Forward scatter was used to distinguish singlets (single cells) from all cells. Live cells were identified as negative for live/dead staining. Live single cells positive for mCherry were identified. Of mCherry-positive cells, CalceinAM-positive and –negative populations were distinguished. Further information on gating strategy is presented in Supplementary Fig. 4.

☒ Tick this box to confirm that a figure exemplifying the gating strategy is provided in the Supplementary Information.
